# Evapotranspiration and favorable growing degree-days are key to tree height growth and ecosystem functioning: Meta-analyses of Pacific Northwest historical data

**DOI:** 10.1038/s41598-018-26681-1

**Published:** 2018-05-29

**Authors:** Yang Liu, Yousry A. El-Kassaby

**Affiliations:** Department of Forest and Conservation Sciences, University of British Columbia, 2424 Main Mall, Vancouver, British Columbia V6T 1Z4 Canada

## Abstract

While temperature and precipitation comprise important ecological filtering for native ranges of forest trees and are predisposing factors underlying forest ecosystem dynamics, the extent and severity of drought raises reasonable concerns for carbon storage and species diversity. Based on historical data from common garden experiments across the Pacific Northwest region, we developed non-linear niche models for height-growth trajectories of conifer trees at the sapling stage using annual or seasonal climatic variables. The correlations between virtual tree height for each locality and ecosystem functions were respectively assessed. Best-fitted models were composed of two distinct components: evapotranspiration and the degree-days disparity for temperature regimes between 5 °C and 18 °C (effective temperature sum and growth temperature, respectively). Tree height prediction for adaptive generalists (e.g., *Pinus monticola*, *Thuja plicata*) had smaller residuals than for specialists (e.g., *Pinus contorta*, *Pseudotsuga menziesii*), albeit a potential confounding factor – tree age. Discernably, there were linearly positive patterns between tree height growth and ecosystem functions (productivity, biomass and species diversity). Additionally, there was a minor effect of tree diversity on height growth in coniferous forests. This study uncovers the implication of key ecological filtering and increases our integrated understanding of how environmental cues affect tree stand growth, species dominance and ecosystem functions.

## Introduction

As early as the 1800s, a nature explorer, Alexander von Humboldt (1769–1859), observed that climate was the primary determinant of global vegetation patterns. At global and regional scales, climate, via affecting plant’s tolerance to abiotic stresses, determines species distribution, form and function^1,2^. Based on plant optimization models^3^, plant height, a trait measured at a higher level of plant integration, is highly sensitive to the environment and often used as a proxy for productivity and fitness^4^. Environmental stressors that limit tree height also act as ecological filters on species diversity, while species diversity has strong, positive and significant effects on tree productivity (a flux in ecosystem functioning) at global scales^5,6^. Such relatedness can be explained by energy-richness theory (i.e., positive correlation between diversity and energy input)^7^. Because tree height reflects species’ carbon gain strategy to secure carbon profit via light capture^8,9^, and further for their growth, survival, reproduction, and competitive ability^10^. As such, tree height is finally able to shape the structure and the composition of forest communities. Globally, temperature has been considered a key environmental correlate of species richness^11^ including in temperate and boreal forests^12^. Species richness is best predicted by climatic variables, such as evapotranspiration (*Eref*, the transfer of water from land surfaces to the atmosphere through turbulence)^13^. *Eref*, also a correlate of productivity, is a vital link between energy, water and carbon cycles^14^ and has a strong correlation with tree species richness in North America^15^. Hence, temperature and evapotranspiration may greatly impact tree height growth and ecosystem functions, whereby tree height growth provides a good surrogate for fitness^16^ and plays an essential role in forest dynamics and resiliency (an epitome for biomass, productivity and species diversity conditions).

Our study location is in the Pacific Northwest region of North America, where the vegetation is primarily occupied by evergreen conifer lineages (Pinophyta as foundation species). Northern ecosystems are characterized by short growing seasons, and plants in such environment are under selective pressures to initiate growth when temperature becomes favorable in the spring, as such temperature should be a key climatic stressor at this region^17,18^. Since gymnosperms are influenced by geographical characteristics at continental scales and do not follow a latitudinal diversity gradient across the North hemisphere^19^, latitudinal gradients, though affecting day length and thus photosynthetic duration, may have little influence on conifer growth, distribution and evolution. Prevailing climates of the Pacific Northwest forests are maritime, varying with latitude and elevation, with rainfall increasing as the elevation uplifts^20^. Nevertheless, strong physiological aridity is another general, primary climatic feature, because water in the soil remains frozen for most of the year, rapidly percolates through sandy soils or its absorption is reduced by surface runoff from mountain slopes^18^ and in snow-dominated forests, growing season moisture is mainly derived from snowpack melt^21^. As such, water availability rather than net precipitation is a key selective force in the Pacific Northwest, which is, in a broad sense, congruent with environmental limitations to the global pattern in plant height^22^. These important environmental conditions co-select for a complex assortment of conifer species, including fir (*Abies* spp.), spruce (*Picea* spp.), pine (*Pinus* spp.), and hemlock (*Tsuga* spp.)^20^. From a micro-evolutionary perspective, conifer species can be classified as adaptive specialists [e.g., lodgepole pine (*Pinus contorta*), Douglas-fir (*Pseudotsuga menziesii*)] and adaptive generalists [e.g., western white pine (*Pinus monticola*), western redcedar (*Thuja plicata*)]^23,24^. Adaptive specialists prompt ample intraspecific differentiation and are subject to strong diversifying natural selection based on temperature and are more vulnerable to maladaptation under climate change^23,24^; by contrast, adaptive generalists lead a more conservative growth habit (e.g., less noticeably biogeographical clines) and result in a relative slow growth^23,24^. The restricted co-tolerance to ecological characteristics, drought versus shade, has been found to be an important facet explaining recent conifer evolution and distribution in North America^25^. The combination of the shade-drought spectrum involves multiple morphological and physiological adaptations and it is a process of differently allocating biomass to dominant height among species (e.g., trade-off between wood density and tree height due to their negative correlation). For instance, compared with drought-tolerant clade [e.g., *Pinus* spp. (high) > *Picea* spp. (moderate) > *Pseudotsuga* spp. (low) > *Abies* spp. (extremely low)], shade-tolerant clades [e.g., *Abies* spp. > *Picea* spp. > *Thuja* spp.] do not cope with drought-induced embolism and have lower wood density^26^. These features allow shade-tolerant species inhabiting dense forests to be taller, better compete for light and rapidly occupy disturbed sites (but not necessarily be early successional species)^26^.

Through this study, we sought to address three commonly invoked questions:

1. *What are the essential environmental drivers for tree height growth?* Among important environmental cues, we expect climatic variables reported in an ample body of literature, such as temperature^12,17,18^ and evapotranspiration (i.e., *Eref*)^14,15^ to weigh more heavily in explaining different height growth and ecosystem functioning at different habitats in the study region.
2. *Do adaptive generalists have stronger environment–trait associations than adaptive specialists?* Since adaptive specialists have a high intraspecific variability and thus have increased abilities to adapt to prevailing ecological gradients^23,24^, we predict that adaptive specialists have better environment–trait matching (i.e., more accurate tree height prediction by the environment) within their ecological breadth.
3. *What is the correlation between tree height growth and ecosystem functions in coniferous forests?* We posit that trees grow faster in high biomass sites because high biomass (a historical factor) is an indicator of good site conditions, thus we expect a positive correlation between tree height and ecosystem functions represented by productivity (a measure of environmental favorability) and biomass. Given a positive relatedness of productivity or biomass with species richness^5,6^, we expect that species diversity has positive effects on tree height growth, but tree diversity may not follow this pattern, as more tree species only represent a wide suitability spectrum of different trees in a particular environment^27^.

Our main consideration of this study lies in constructing niche models to explore climate-growth relations instead of height–yield trajectory prediction by species at sites. In light of phylogenetic niche conservatism (i.e., the tendency of lineages to be restricted to their ancestral niche, potentially encouraging *in situ* radiation) and tropical niche conservatism hypothesis (i.e., species in regional assemblages in colder or drier climates are more closely related to each other)^28^, we accentuated species attributes as outcomes of ecological processes in the Pacific Northwest and dealt with all conifer species as one single clade (i.e., phylogenetically clustered together). For modeling simplicity, we focused on the allometry of conifer trees at the sapling stage (age under 25 years) using a monotonically increasing function and differences in early height growth (e.g., in *Picea* spp.) are ascribed to strong diversifying selection^29^.

## Material and Methods

### Tree height data compilation

**Figure 1:**
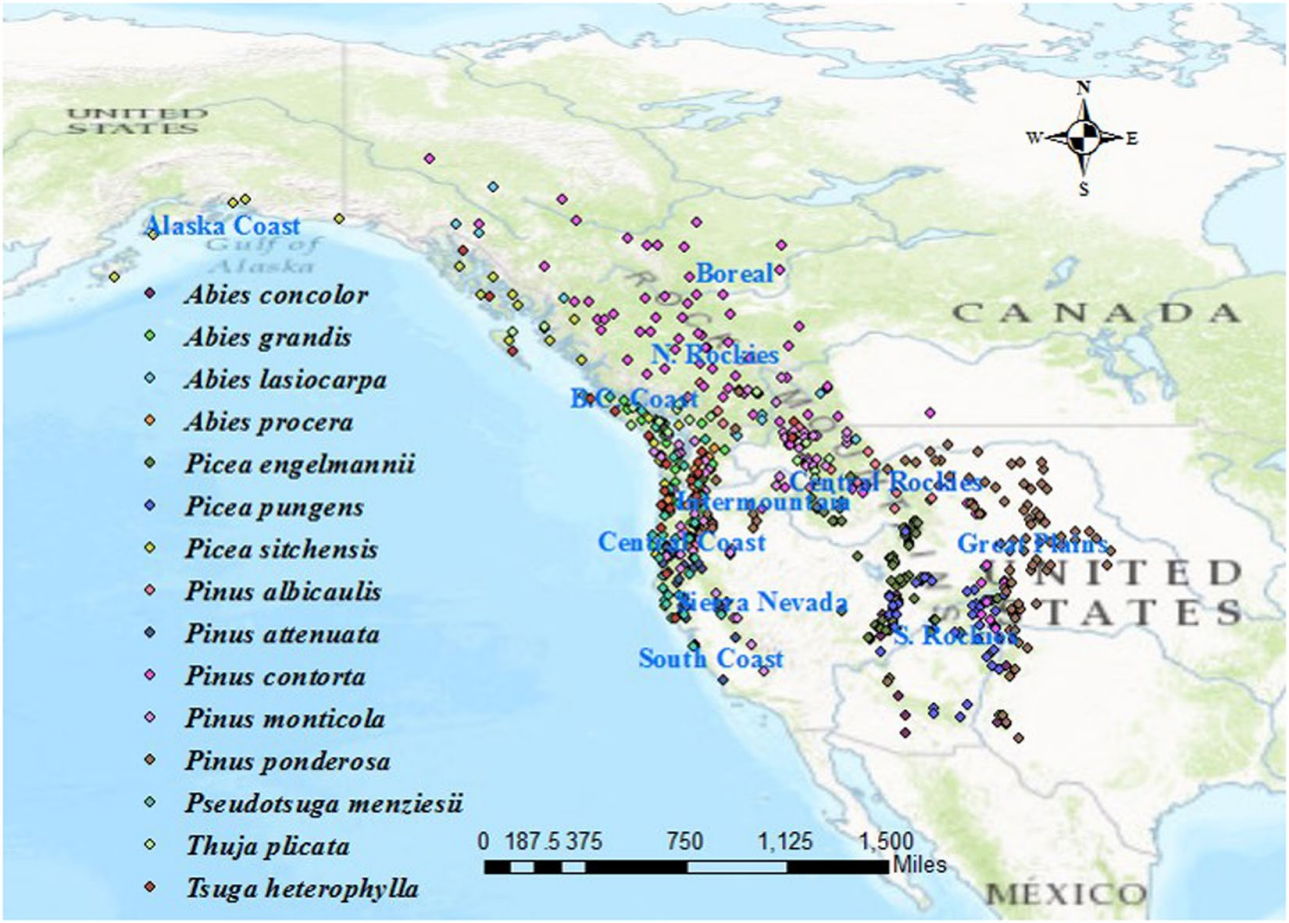
Location of conifer populations (colored circles for different species) used in this study. Test populations cover main ecozones in the Pacific Northwest, including areas of Alaska Coast (Southern Alaska and Alaska panhandle), BC Coast (coastal British Columbia), Central Coast (coastal Washington, Oregon and northern California), South Coast (coastal California south of San Francisco), Boreal (the Yukon, British Columbia and interior northern Alberta), N. Rockies (interior southern British Columbia), Central Rockies (Rocky mountains in Idaho, Montana and Wyoming), S. Rockies (Rocky mountains in Arizona, Colorado, New Mexico, and Utah), Intermountain (interior Oregon and Washington), Sierra Nevada (inland California), and Great Plains (foothills east of the Rocky Mountains). General climate features in aforementioned ecozones^50^: late summer drought is typical throughout most of the interior of the region, particularly for parts of the Intermountain and the Boreal, but along the BC Coast and in the N. Rockies, water is not a selective stressor because ample precipitation fully recharges the soil profile and transpiration does not exhaust this reservoir during the growing season. The mountains of Central and S. Coasts are buffered from temperature extremes, whereas diurnal variation increases with elevation and with movement inland. High evaporative demand in the summer is typical in Sierra and Nevada, and Central and S. Rockies regions remain cool throughout the year. The map was generated on ArcMap ver. 10.5.1 at http://desktop.arcgis.com/en/arcmap/.

Tree height data for widespread keystone forest tree species, conifers, in western North America were compiled opportunistically from previous studies, listed in Note S1. All pooled tree height values were converted to meters prior to use. The final dataset comprised an average of *c*. 50 conifer tree individuals per population (N.B. missing information in data source excluded from this calculation) sourced from a total of 616 geographically diverse populations in 15 coniferous species covering ecosystems from the boreal to the subtropics (Fig. 1, generated on ArcMap ver. 10.5.1 at http://desktop.arcgis.com/en/arcmap/). The native ranges of the 15 species were displayed on the map (Fig. S1) and potential selective drivers for such geographic distribution were summarized in Table S1. The data were compiled from 21 studies, in which common garden experiments were carried out for multi-site provenance tests (i.e., individuals from the same set of populations are planted along a wide climatic range) during the 1962–2004 period. These provenance trials have revealed that phenotypic responses are often based on phenotypic plasticity and local adaptation. As we used average tree height in each plot, their observed differences among sites are assumed to be largely environmentally-based.

### Climate datasets and selection of representative variables

Based on sapling planting (tree age under 25 years) and sampling year, climate normals for each population for the periods 1961–1990 or 1981–2010 were accordingly obtained using ClimateNA ver. 5.40^30^. ClimateNA is a standalone software application^30^ that extracts and downscales gridded (4 × 4 km) monthly climate data for the reference normal period (1961–1990) from PRISM^31^ and WorldClim^32^ to scale-free point locations. It also calculates more than 200 monthly, seasonal and annual climate variables. The downscaling is accomplished through a combination of bilinear interpolation and dynamic local elevational adjustment. ClimateNA also uses the scale-free data as baseline to downscale historical and future climate variables for individual years and periods between 1901 and 2100. We projected climatic variables for the pooled sites from forest inventory data using a reference climate normal period (1961–1990), preceding pronounced climate warming of the last *c*. 25 years. The pre-warming climate is likely to more closely reflect the historical conditions to which test populations may be locally adapted.

To extract a suite of important climatic data from 218 annual, seasonal and monthly variables that can most explain the tree height variation, we performed partial least squares regression (PLSR) using the PLS package in R ver. 3.3.2^33^. This multivariate regression method combines features from Principle Component Analyses and multiple regression and adopts a machine-learning algorithm in model optimization (as previously described^34^). We employed Leave-One-Out cross-validation for the whole data and selected principle components when Root-Mean-Square Error of Prediction did not show a significant decrease. Loadings give correlations between scores and original variables and were used as a criterion for climatic variable selection (cutoff = 0.4 on main principle components). Height growth potential for original studies was calculated and used as the response variable in the PLSR process.

### Niche-based model for height-age adjustment

**Figure 2:**
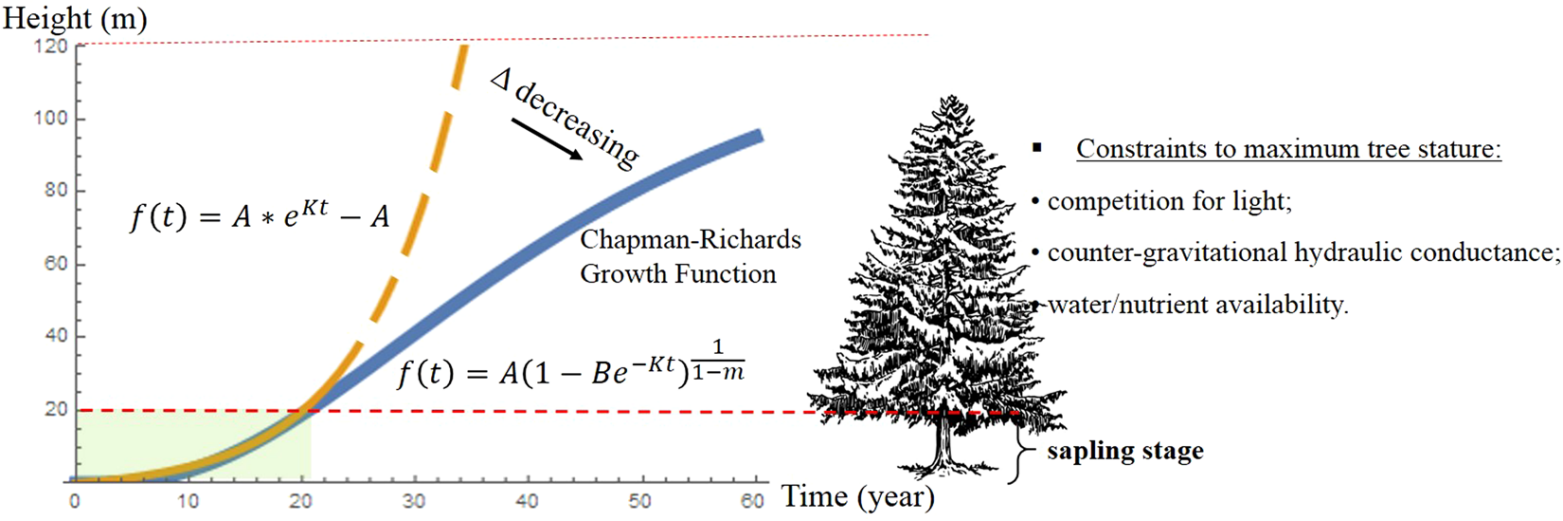
Conceptual illustration of tree-growth trajectory models. Solid and dashed curves are fitted using Chapman-Richards (*A*, *B*, *K* > 0 and 0 < *m* < 1) and exponential functions, respectively. At the sapling stage (light green-shaded area, indicating our data range), the two curves are overlapped. Due to complex constraints to tree stature imposed after sapling stage (e.g., thickened and gnarled branches and flattened canopy), tree height increment decreases and Chapman-Richards function particularly simulates this trend. Tree silhouette image reprinted with permission of source at https://pixabay.com/.

The model was established on the nonlinearity framework used in the generalized Chapman-Richards function, a standard for height–growth relationships^35^ with modifications (Fig. 2). We pooled conifer sapling height data spanning 25 years of age and growth fitted in exponential curves was deployed. Succinctly, the constant parameters in the general nonlinear model (Eqn. 1) were replaced by climatic functions (θ’s in Eqn. 2) which represented nonlinear parameter estimates. Climatic functions were built using either annual or seasonal variables.

1 $$y=f(x,\,\theta )+\varepsilon $$

2 $$f(x,\,\theta )={\theta }_{1}{e}^{{\theta }_{2}x}-{\theta }_{1}$$

where, *x*-variable is the fixed-effects term (i.e., age) and the error terms (ε’s) are the only random variables. We assumed that the errors are normal and i.i.d. (i.e., independent and identically distributed), suggestive of a simple error structure with equal variances and zero covariances. We conducted model fitting using the Full Information Maximum Likelihood estimation method under the PROC MODEL procedure in SAS 9.4^36^. Eight-year-old was the average age of the measurements, also very accurate and informative to the climate-growth relationship, and adopted as the standard age throughout this study, unless otherwise indicated.

To explore which climatic variables explain best the height growth and greatly contribute to ecosystem functioning, all possible combinations of selected annual and seasonal climatic variables (n = 12, selected as per PLSR) were used to develop climatic functions. As an approximation, the curve was vertically shifted up by θ_1_ unit (i.e., no intercept adjustment in Eqn. 2) and then log-transformation was applied to Eqs 2, 3. We generated a total of 4,096 subsets linear regressions $$[{\rm{i.e.,}}\,N={\sum }_{k=0}^{12}(\begin{array}{c}12\\ k\end{array})={2}^{12}]$$ for Equation 3.

3 $$\mathrm{Ln}\,(\hat{y})=\,\mathrm{Ln}\,({\theta }_{1})+{\theta }_{2}x$$

In addition, we carried out second derivative and integral for one selected model under different climatic scenarios (current, 2025s, 2055s and 2085s), which tells how rapidly the rate of change of height growth is changing and how different cumulative height growth is in time series under climate change, respectively. The values of the climatic variables used in the model were averaged for temporal comparisons throughout study sites showcased in Fig. 1.

### Model selection and validation

To evaluate the reliability of the model to different species, we utilized a ten-fold cross-validation method^37^. Model bias and Root-Mean-Square Error (RMSE) for each species were respectively calculated (Eqs 4 and 5). The bias and the RMSE correspond to the error associated with the prediction for all populations and one single population, respectively. Positive (or negative) bias represents the model underestimated (or overestimated) the observations.

4 $$bias=\frac{{\sum }_{i=1}^{n}({y}_{i}-{\hat{y}}_{i})}{n}$$

5 $$RMSE=\sqrt{\frac{{\sum }_{i=1}^{n}{({y}_{i}-{\hat{y}}_{i})}^{2}}{n}}$$

where, ${y}_{i}$ and ${\hat{y}}_{i}$ are observed and estimated values for tree population *i* and *n* is the total number of tree populations.

To select models with more parsimonious structure, we computed estimates of the magnitude of the typical residual from the fitted model using the Akaike’s Information Criterion (AIC), RMSE and R-squared. Scripts for cross-validation and all subsets regression functions were written on R ver. 3.3.2^33^.

### Ecosystem functions extracted from forest inventory data

To explore our third principal question regarding the relationship between tree height and ecosystem functioning, we aggregated 80,119 plots (i.e., average of subplots within the same plot) using the US Forest Service’s Forest Inventory and Analysis (FIA) data in the Pacific Northwest region. The FIA data cover tracts of coniferous forests but the boreal biome considered in our niche models (i.e., British Columbia and Alberta, Canada) is not within its boundary; specifically, 16 US States were included: AK, WA, OR, CA, ID, NV, MT, WY, UT, AZ, CO, NM, ND, SD, NE, and KS [https://www.fia.fs.fed.us/, last accessed in February 2017] (Fig. S2). For inclusion in the analysis, only plots with 70% tree species covered by conifers were retained (totaling 77,676 plots) and data from the same plot sampled more than twice over time were averaged. While our selection criteria ended up rendering a total of *c*. 44,000 plots for relationship studies of tree height with productivity, biomass and alpha tree diversity (i.e., the number of trees within a sampling plot), only 301 plots were available for alpha species diversity – tree height study. It should be mentioned that tree or species diversity incorporates two components: tree or species richness and their distribution of abundance as a measure of evenness. According to the FIA manual, species richness denotes the total number of species including all trees, shrubs, herbs, grasses, ferns, and fern allies (horsetails and club mosses) occurring on the plots. Of the plots, the predicted height at 8 years old ranged between 0.46 and 4.77 m. The R code for the data extraction from the FIA was provided in Appendix S1.

### Data availability

Sources of the compiled meta-data are listed in Note S1 and can be extracted from US Forest Service’s Forest Inventory and Analysis (see the FIA manual for details: Phase 2 ver. 6.1.1 and Phase 3 ver. 6.0.1). All relevant data contained within this article are available from the corresponding author upon reasonable request.

## Results

**Figure 3:**
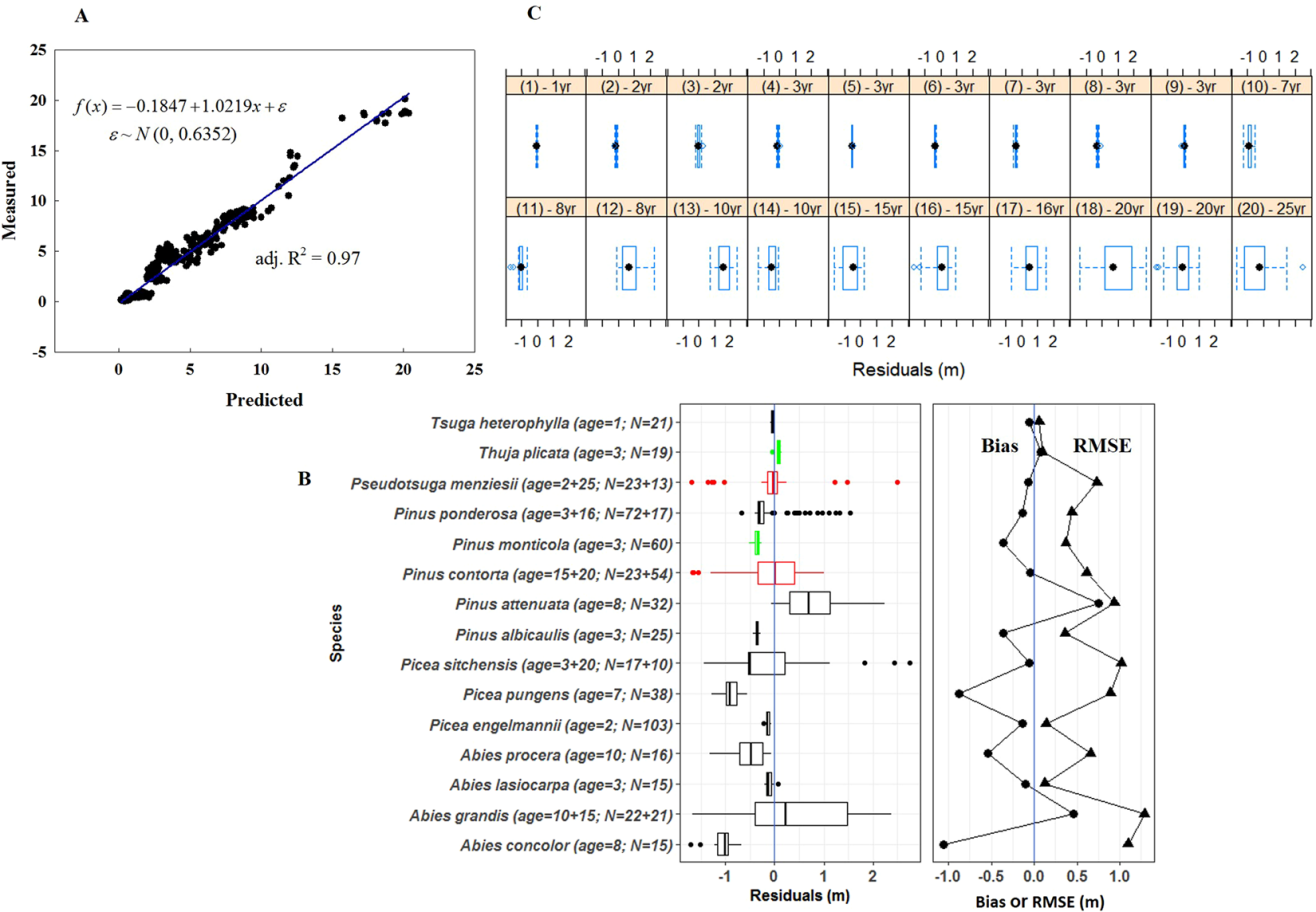
Linear relationship between measured and predicted height at the age of 8 years old (**A**) and residual, bias and RMSE (m) calculated by species using the best-fit model (**B** and **C**). The best-fit model was described in Table 1a. In (**B**) box plots in green and red indicate adaptive generalists and specialists, respectively; overall bias and error are −0.1287 (indicative of overestimation) and 0.6526 m, respectively. Tree ages and sampled population sizes, *N*, are given within parentheses in the vertical axis. In (**C**) residuals for each species by age were displayed in box plots. Species age in year (yr) was given above each panel and species in the 20 panels are *Tsuga heterophylla* (1), *Picea engelmannii* (2), *Pseudotsuga menziesii* [(3) and (20)], *Abies lasiocarpa* (4), *Picea sitchensis* [(5) and (18)], *Pinus albicaulis* (6), *Pinus monticola* (7), *Pinus ponderosa* [(8) and (17)], *Thuja plicata* (9), *Picea pungens* (10), *Abies concolor* (11), *Pinus attenuate* (12), *Abies grandis* [(13) and (15)], *Abies procera* (14), and *Pinus contorta* [(16) and (19)].

The regression via an exponential function well summarized the original height data of conifer trees under 25 years (*R*^2^ = 0.92) (Fig. S3). Using growth potential as the response variable, loadings in PLSR indicated that the most critical annual climatic variables were degree-days below 18 °C [*DD_18*] and degree-days above 5 °C [*DD5*] (Fig. S4); scores plot showed no apparent indication of grouping or outliers, suggestive of a good coverage of height ranges (Fig. S5). Pairwise correlations for monthly climatic variables showed that Hargreaves reference evaporation [*Eref*] had a positive correlation with other variables (except with precipitation [*PPT*]) and bore a non-overlapping feature between first and second half of the year (Fig. S6). Based on these results, we finally selected 12 key climatic variables, including four annual variables (*Eref*, *DD_18*, *DD5* and a commonly used variable, mean annual temperature [*MAT***]**) and eight seasonal variables for *DD_18* and *DD5*. Best-fit growth functions showed that *DD5*, *DD_18* and *Eref* contributed most to the selected fitted models (Table 1) and we finally chose the best annual climatic variable-based model for the subsequent analyses (Table 1a). There was a high correlation between predicted-measured tree height (*R*^2^ = 0.97, Fig. 3A) and we noted that height had linearly high significant correlations (*R*^2^ > 0.70) with *DD5*, *DD_18* and *MAT*, though low (*R*^2^ = 0.22) with *Eref* (Fig. S7). This suggests that paired linear correlation between height growth and *Eref* alone failed to reflect the true contribution of *Eref* to the height–growth pattern.

**Table 1:** Best-fit growth functions and coefficients used in selected models (full model in Eqn. 2).

Best-fit function	Parameter estimates	Goodness-of-fit statistics		
		Pseudo *R*^2^	RMSE	AIC
**(a) Key annual climatic variable-based model**				
θ_1_ = a_10_ + a_11_*DD5 + a_12_*DD_18 + a_13_*Eref + ɛ_1_ θ_2_ = a_20_ + a_21_*DD5 + ɛ_2_	a_10_ ~ N(2.801085, 0.2903), a_11_ = 0.061629, a_12_ = −0.02025, a_13_ = −0.15363 a_20_ ~ N(0.099673, 0.00357), a_21_ = −0.00037	0.9703	0.6558	1234.25
**(b) Key seasonal climatic variable-based model**				
θ_1_ = a_10_ + a_11_*DD_18_sp + a_12_*DD5_sp + ɛ_1_ θ_2_ = a_20_ + a_21_*DD5_sp + ɛ_2_	a_10_ ~ N(1.720139, 0.2887), a_11_ = −0.05123, a_12_ = 0.217443 a_20_ ~ N(0.10765, 0.00316), a_21_ = −0.00361	0.9657	0.7047	1322.01
**(c) Key climatic variable-based model (annual** + **seasonal)**				
θ_1_ = a_10_ + a_11_*DD5 + a_12_*DD_18 + a_13_*Eref + a_14_*DD_18_sp + a_15_*DD5_sp + ɛ_1_ θ_2_ = a_20_ + a_21_*DD5 + a_22_*DD5_sp + ɛ_2_	a_10_ ~ N(2.959389, 0.4974), a_11_ = −0.09031, a_12_ = −0.03432, a_13_ = −0.12187, a_14_ = 0.079409, a_15_ = 0.737145 a_20_ ~ N(0.089493, 0.00538), a_21_ = 0.001929, a_22_ = −0.01123	0.9715	0.6427	1212.50

Note: *DD5*, and *DD_18*, *Eref*, *DD5_sp*, *DD5_at*, *DD_18_sp*, and *DD_18_at* are scaled down by 100 in units in the functions.

Models are only applicable to the locations with *DD5* larger than 100 (original unit).

Parameters estimates are significant at α = 0.05 using *t*-test; otherwise, we underscored the parameter in the function (see Table S2). N(µ, σ) gives normal distribution with mean µ and standard deviation σ. All errors (ɛ’s) are assumed to be identically and independently distributed (i.e., “i.i.d”).

Pseudo *R*^2^ = 1 − SSE/SST_corrected_; ${\rm{Root}} \mbox{-} {\rm{Mean}} \mbox{-} {\rm{Squared}}\,{\rm{Error}}\,({\rm{RMSE}})=\sqrt{\frac{1}{n-p}\sum _{i=1}^{n}{({y}_{i}-{\hat{y}}_{i})}^{2}=\sqrt{\frac{SSE}{n-p}}}$; Akaike’s Information Criterion (AIC) = −2ln $\hat{L}$ + 2p = n * [log(2π) + log(${s}_{n}^{2}$) + 1] + 2p [n: total number of observations (or sample size); p: number of estimated parameters (including intercept and variables); log $\hat{L}$: log likelihood; ${s}_{n}^{2}$: sum squared residuals weighted by sample size].

To get the best model, we tested whether adding the climate variable from the second-best model significantly improved the fit (evaluated by ΔAIC > 2; N.B. AIC and Leave-One-Out cross-validation are asymptotically equivalent (for other good models, see Table S2).

**Figure 4:**
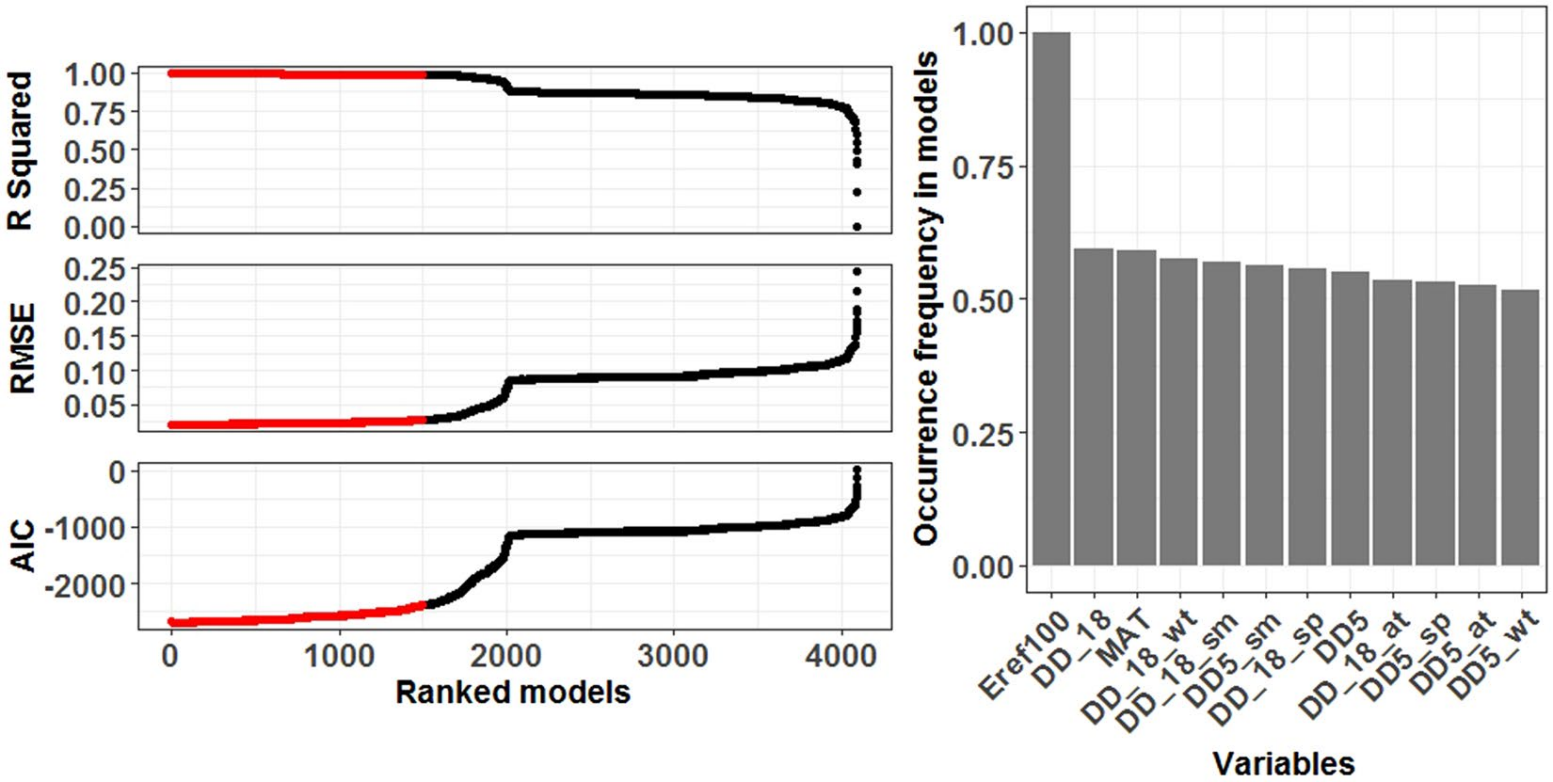
Values of AIC, RMSE and *R*^2^ for the models using a random combination of key 12 climatic variables (4,096 models ranked by AICs in ascending order) and occurrence frequency of all variables in the top 1,500 models. N.B. *Eref*100 denotes *Eref* scaled down by 100.

**Figure 5:**
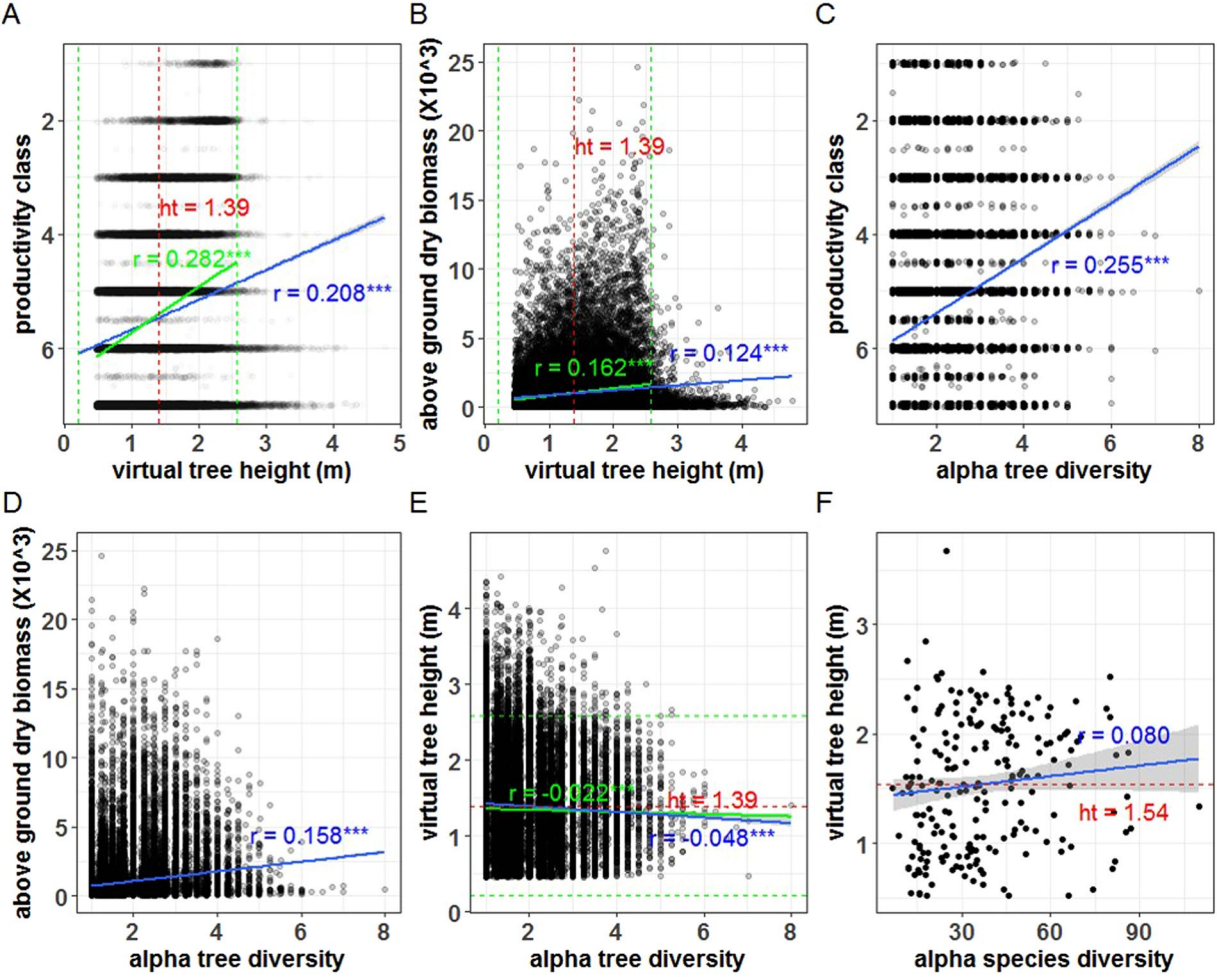
Relatedness of tree height with productivity and above-ground biomass (**A**,**B**), effects of alpha tree diversity on productivity, biomass and height growth (**C**,**E**) and effects of alpha species diversity on height (**F**). Average height (ht) is given in panels in red (also in red dashed line); linear regressions and Pearson correlations (*r* values) for the entire and 95% data (i.e., mean +/− 2*std) are fitted and calculated, marked in blue and green, respectively. Dashed green lines set the boundary of 95% data. Three asterisks (***) indicates very significant at *p* < 0.001 and the regression line of a non-significant model (*p* ≥ α = 0.05) is not displayed. Alpha tree (species) diversity is the total number of trees (species) within a sampling plot. FIA productivity classes estimate the potential growth for plots to produce wood in cubic feet/acre/year based on the culmination of mean annual increment of full stocked natural stands and are as follows: 1 = 225+, 2 = 165–224, 3 = 120–164, 4 = 85–119, 5 = 50–84, 6 = 20–49, and 7 = 0–19. Aboveground dry biomass = dry biomass in the merchantable bole + dry biomass in the top and limbs of the tree + dry biomass in the tree stump + aboveground dry biomass of saplings + aboveground dry biomass of woodland tree species (i.e., DRYBIO_BOLE + DRYBIO_STUMP + DRYBIO_TOP + DRYBIO_SAPLING + DRYBIO_WDLD_SPP).

As predicted, tree height growth in adaptive generalists was better projected than in specialists, albeit small residuals at the juvenile sapling stage (compare residuals in Fig. 3B). There was no direct, positive relatedness between age and prediction accuracy (see the plot from original data in Fig. S3). Given comparison at the same age, for instance, two-year *Pseudotsuga menziesii* had large variance (compare Fig. 3C-3 with Fig. 3C-2 and even with Fig. 3C-4~9). Furthermore, of the 4,096 models constructed via the combinations of 12 selected climate variables, the top 1,500 models (ranked by AIC) had *Eref* as variable (frequency = 1.00), contrastingly higher than that of the other 11 variables [frequency ∈ (0.50, 0.62)] (Fig. 4). Tree height growth was positively correlated with productivity (*r* = 0.282, *F*_*1*, 42724_ = 3705, *p* < 2.2e-16) and above ground dry biomass (*r* = 0.162, *F*_*1,42228*_ = 1138, *p* < 2.2e-16) (Fig. 5A,B). Alpha tree diversity had positive effects on productivity (*r* = 0.255, *F*_*1,44061*_ = 3060, *p* < 2.2e-16) and above ground biomass (*r* = 0.158, *F*_*1,43547*_ = 1111, *p* < 2.2e-16) (Fig. 5C,D), but tree diversity had significantly but negatively weak correlation with height growth (*r* = −0.022, *F*_*1,42575*_ = 20.62, *p* < 5.6e-06) (Fig. 5E). Alpha species diversity had insignificantly positive influence on height growth (*r* = 0.080, *F*_*1,211*_ = 2.38, *p* = 0.125) (Fig. 5F). Additionally, in the context of climate change, growth speed and height yield may increase in the entire study region (Fig. S8).

## Discussion

Across Western North America north of Mexico, conifers have become dominant in regions characterized by conditions that angiosperms tolerate poorly, such as, extreme cold, severe aridity, and nutrient-poor soils^18^. By weaving together a series of disparate threads encapsulating environmental limitations to native species ranges, past common garden experiments conducted in conifers and exceedingly large forest inventory data in the Pacific Northwest, we have documented how ecological filtering shapes tree height growth trajectories and influences ecosystem functions. Our results provided reasonable insights to the three questions we put up and noteworthy results include: (1) the essential ecological drivers for tree height growth primarily include evapotranspiration and degree-days for temperature regimes between temperature sum (cumulative temperatures ≥5 °C) and growth (≤18 °C) (Table 1); (2) adaptive generalists are better projected than specialists via a niche model (Fig. 3); (3) there is positive relatedness of height growth with productivity and biomass (Fig. 5A,B) and it is the diversity of species rather than trees that has positive effect on tree height growth in coniferous forests (Fig. 5E,F).

Although plant growth is limited by three primary factors, water, energy and nutrient availability, competition with angiosperms may restrict conifers to habitats having low productivity or high disturbances (e.g., fire-prone sites^38^), such that water and energy may be the true leading drivers for conifer distribution in the absence of soil considerations. Evapotranspiration, *Eref*, representative of the land-atmosphere coupling, is a multifaceted, keystone climate variable that uniquely links the water cycle (i.e., evaporation), energy cycle (i.e., latent heat flux or the amount of energy used in evaporating water), and carbon cycle (i.e., transpiration-photosynthesis tradeoff)^39–41^. It is linearly correlated with biomass production when being used as a function of net radiation^42^ and the leading climatic predictor in species diversity assessments^43^. Carbon balance theory indicates that tree height growth is determined by the difference between carbon assimilation (photosynthesis) and respiration (in stems, foliage and roots)^44^. Our model bolsters the essential role of *Eref* and captures the causal effect of tree height growth and its feedback to ecosystem functioning (Table 1 and Fig. 4). It should be mentioned that *Eref* used in this study is Hargreaves reference evaporation, representing the evaporation potential^45^, rather than the actual evapotranspiration at locations.

In addition to *Eref*, spring temperature sum (*DD5_sp*), representative of spring energy availability, is another important climate variable (Table 1). Warm air temperature in the spring initiates evapotranspiration and photosynthesis (because of little light in the winter), and climatic triggers for spring budburst are apparently crucial to tree height growth. A combination of high temperatures during the growing season and low winter-spring precipitation of the previous year can explain much of the variation in conifer growth rates in the southwest US^46^. Growth of conifers (e.g., *Picea* spp.) decreases as tolerance to extreme cold increases with latitude or local tolerance of deep snow pack and short growing seasons increases with elevation^47,48^. As environmental cues experienced within one season could affect growth in the following season and annual variables are more stable and accurate over years, it is biologically reasonable, particularly in a long run, to rely on the model fitted by key annual instead of particular seasonal climatic variables for dynamic analysis throughout time.

These findings supplement previous scientific attempts conducted in conifer species in the same region. For instance, the height of 20-year *Pinus contorta* in British Columbia and Yukon, Canada was projected by a single universal response function using annual mean temperature (*MAT*), annual heat-moisture index and geographic variables^49^. The distribution of 15 native coniferous species was modelled by a generic process-based growth model using climate-based variables reflecting deficits in the soil water balance and departures from optimum temperatures in the summer^50^. The current endeavor to this topic employs an ecologically sensible model to explicitly demonstrate which climatic variables essentially drive height growth in a collection of major conifer species across the Pacific Northwest and to what extent it contributes to ecosystem functions. On the regards of study scale and perspectives, this study advances our holistic understanding for the pattern and relationship among tree height growth, climate and ecosystem functioning.

Trees have the capacity to fill gaps resulting from canopy openings and shade-tolerant species are particularly efficient in this attribute, such that we postulated that increasing tree diversity should facilitate tree height growth, as the species richness–tree height relationship is in a positive manner. Our results, however, did not support this extrapolation (Fig. 5E). We think that this lack of congruency has two-faceted reasons. First, height growth of a particular conifer species is highly dependent on age and site conditions [including climate, soil properties (e.g., nitrogen for boreal forests), competition intensity from neighboring trees, etc.] Large crown dimensions are also beneficial to tree height growth. Our model solely considered effects of climatic factors on tree height growth during the sapling period, while tree diversity may significantly impact tree height growth when tree competition or interaction intensifies and affects tree height growth. Moreover, in contrast with structurally distinct layers in many forest types, coniferous forests have only two distinct layers, namely, canopy layer made up of trees growing close together to a uniform height (20–30 m) and undergrowth layer having very little vegetation due to a low amount of sunlight and poor soil fertility. This indicates that tree diversity in coniferous forests plays a less influential role in tree height growth than in other types of forests. It is more likely that there is a positive effect of tree diversity on maximum tree height than on height growth of saplings.

Second, species diversity is often strongly correlated with the current environment, especially with climate [e.g., water and energy^51^], indicative of important roles of current ecological limits to species diversity. Besides climate, diversity patterns in regional assemblages also hinge on long-term speciation and extinction processes and evolutionary factors (e.g., diversification, time for speciation effect, niche conservatism), and correlations between species diversity and current climate may be therefore due to its resemblance to past climate and the extent to which current diversity patterns are legacies of past conditions [e.g., temperature and precipitation^52^]. The Cenozoic extinction events (peaks in *c*. 29, 16, and 7–5 Myr) explain young crowns and low diversity of living gymnosperm lineages^53^ and this fact partially accounts for little effect of tree diversity on tree height growth (Fig. 5E) and no strong effect on productivity and biomass (Fig. 5C,D). In addition, tree diversity in our studied sites ranged between 1 and 8 with median at 2.5 (see the horizontal axis range in Fig. 5E), and low net tree diversity may reduce its cascading effects on tree height growth.

Albeit minor effects of tree diversity on tree height growth, climate change may greatly impact height growth. Different sensitivity of tree height growth at different sites to changes in climate reveals how ecosystem responses to climate change will be spatially variable. Climate change is increasing water stress as a result of increasing evaporative demand, altered rainfall and earlier snowmelt. Warming is considered most important to seasonal soil water balance due to changes in snowpack dynamics and/or *Eref *^46^. If climate change results in rising CO_2_, increased moisture availability and warming, production and growth may increase, evidenced by an example in temperate-maritime forests of the Pacific Northwest^54^. Warming in the autumn likely delays cessation and extend the growing season particularly at high latitude and elevation locations. However, if climatic drivers are drought (or moisture deficit and warming) in temperate or boreal forests, decreased growth, reduced biomass and increased mortality could be expected^55–57^. Many recent forest declines are triggered, physiologically, by drought stress and through weakening tree defenses, makes trees more susceptible to be attacked by insects or pathogens^56,58^. As such, varying water deficits appear to be the primary drivers of variation in tree recruitment and mortality and our model included indices of warming and drought (i.e., *Eref*, *DD5* and *DD_18*; also see Table 1 for model details).

Species diversity may mitigate climate change impacts on species diversity itself (because species diversity provides a buffer to maintain ecosystem functions in the presence of environmental variability, in that different species respond differently to environmental fluctuations) and ecosystem functioning via the positive relationship between diversity and ecosystem functioning. Low diversity of conifer species makes conifer tree height growth more vulnerable to be influenced by climate change. Our prediction evinces that height growth speed and yield at the sapling stage may increase in the Pacific Northwest in the future (Fig. S8), and this indicates that climate change, via attenuating a prevalent driver, cold temperature, promotes tree height growth, however, we should be circumspect when interpreting this. Because climatic effects on final tree size and ecosystem functioning have not been evaluated. Apparently, mounting clues have directed our attention to the drought-induced stress or mortality that may become a new inciting limitation to tree height growth in future scenarios.

Furthermore, climate change facilitates the selection of adaptive strategies at the community level. Adaptive generalists are more plastic, can use a large number of environmental resources and survive better via changing architectural, physiological, or phenological traits under varied climate conditions^59^. Natural selection would favor a generalist strategy in populations that experience spatially high variability in resource availability possibly due to population-level variation in competitive pressures^60^ or some local characteristics, such as, edaphic factors^61^ or biotic and abiotic factors^62^. By contrast, adaptive specialists are much more sensible to global change and easier to become non-adapted when environmental conditions have largely changed. Specialization can be particularly advantageous if the costs of being a generalists are high, such as, specialization allows for higher resource use efficiency^63^. This prompts that, in response to year-to-year changes in environmental indicators of resource availability, species dominance in communities will change and generalist strategies would be most common in species. There may be less communities dominated by adaptive specialists and adaptive generalists may invade most of habitats, thus leading to biotic homogenization (i.e., strong resemblance between communities with different types of habitats). Such species dominance will have cascading influences on ecosystem functioning. According to our results, decreasing species diversity via excluding adaptive generalists will lower productivity and above ground biomass (Fig. 5C,D).

The lineage feature of tolerance to drought stress may put forth another important aspect that affects species dominance at the community level. Although *Pinus monticola* and *Thuja plicata* are adaptive generalists and *Pinus contorta* and *Pseudotsuga menziesii* are specialists, *Pinus* spp. is more tolerant to xeric environments and thus conducive to being retained due to climate change. This indicates that predicting how species dominance changes as climate changes should consider the sensitivity of species and their capacity of tolerance to drought stresses. Moreover, even given a specific species, a warming climate may be relatively more beneficial, if any, to northern populations than to southern counterparts. Because general knowledge has informed us of superior growth attributes for southern populations relative to northern ones. This suggests that species dominance in communities may be affected by population origins in the context of global warming.

Finally, we again point out that one caveat to our findings is that our niche model for tree height growth was based on provenance trials at the sapling stage in the entire Pacific Northwest region, while the correlation between tree height–ecosystem functioning was tested using western US forest inventory data, in which the boreal ecozone was not covered. Although tree diversity has a little impact on tree height growth in the conifer-dominated region, species composition has important consequences to species diversity and ecosystem function reported in boreal forests^64^ (also see Fig. 5F), where compositional shifts from late- (e.g., *Picea* spp.) to early-successional conifers (e.g., *Pinus* spp.) and deciduous broadleaves (e.g., *Populus* spp.) due to increased fire frequency associated with climate change. This may be propelled by rising atmospheric CO_2_ and warming^64^. As such, atmospheric CO_2_ concentration may be another important factor in modeling tree height growth and ecosystem functions, as forest types affect the capacity of carbon sequestration and are further correlated with productivity. It should be mentioned that woody species height is not only determined by habitat productivity, but also depends on the time and interval of disturbances^65^. It is therefore necessary to consider the dynamic processes under non-benign conditions such as species competition, effects of CO_2_ enrichment, interactions among drought, fire, bark beetles, pathogens, climate change, anthropogenic interferences, etc. How these disturbance agents affect forest disturbance regimes via direct, indirect and interaction effects requires more comprehensive studies.

## Conclusions

Through a niche model (inclusion of annual, seasonal climate variables, or a combination thereof) developed for an assemblage of major conifer species across the Pacific Northwest region of North America, tree height growth, tree productivity, above-ground dry biomass, tree diversity, and species richness were lumped and contrasted throughout in this study. Implicit climate factors for height growth in the Pacific Northwest comprise evapotranspiration and degree-days above 5 °C but below 18 °C. The niche model verified that the height trait for adaptive generalists (e.g., *Pinus monticola*, *Thuja plicata*) can be more accurately predicted than that for adaptive specialists (e.g., *Pinus contorta*, *Pseudotsuga menziesii*). There are significantly positive correlations of tree height growth with productivity and biomass; in addition, high species richness alludes to tall tree height. Although effects of tree diversity on productivity and biomass are positive, such effects are limited on tree height growth in coniferous forests. This study updates our knowledge and formulates novel perspectives regarding how the environment shapes the functional trait and further impacts ecosystem functioning in the region of the Pacific Northwest.

## Electronic supplementary material

Appendices S1-S3
