# Supplementary material for: Evapotranspiration and favorable growing degree-days are key to tree height growth and ecosystem functioning: Meta-analyses of Pacific Northwest historical data
Source: Sci Rep. 2018 May 29;8:8228. doi: 10.1038/s41598-018-26681-1 (PMC5974028; doi:10.1038/s41598-018-26681-1)
Supplement: Supplementary file 1 — Appendices S1-S3 [file 41598_2018_26681_MOESM1_ESM.pdf]

SUPPORTING INFORMATION

Evapotranspiration and favorable growing degree-days are key to tree height growth and ecosystem functioning: Meta-analyses of Pacific Northwest historical data

Yang Liu, Yousry A. El-Kassaby

Appendix S1: R code for the FIA data compilation
Appendix S2: Supplemental notes and tables
Appendix S3: Supplemental figures

```
General Description:

According to the FIA manual, each plot has a total area of 672 m^2 and consists of four subplots
arranged in a cluster, with one central plot and three peripheral plots spaced 120 degree apart in
a 36.5 m radius circle around the central plot. Productivity class was extracted from COND files,
biomass and alpha tree diversity (i.e., the number of species within a plot) from TREE files,
species richness from VEG_PLOT_SPECIES files, and geographical coordinates from PLOT files. After
a suite of filtering for NA values in the eligible plots and predicted height smaller than zero
for ages at 3, 8, 15 and 20 both before and after corrections using the Measured-Predicted
function, a total of c. 44,000 plots (only 301 plots were available for species richness-height
study) were rendered.
We did not use "natural stand" (i.e., plots that forested and lack any evidence of artificial
regeneration, such as, wooded pasture, windbreaks) as a coerced filtering condition, because there
are many plots without stand origin information, and the subsequent calculation used fully stocked
natural stands (nature stand factor was considered; e.g., see tree productivity classes).

#All data will be deposited at Dryad Digital Repository; alternatively, they can be directly
requested from corresponding author.

rm(list = ls())

setwd("PathToDataDirectory")#all original data are archived in the working directory.
getwd()

library(plyr)

data_SPCD_All=read.csv("Tree_species_code.csv",header = T)
#this can be extracted from Appendix F in FIA Phase 2

L1 = list.files(pattern="*_TREE.csv")
L2 = list.files(pattern="*_VEG_PLOT_SPECIES.csv")
L3 = list.files(pattern="*_PLOT.csv")
L4 = list.files(pattern="*_TREE.csv")
```

```
L5 = list.files(pattern="*_COND.csv")

###step 1: get alpha tree diversity
data_all1 = lapply(L1, function(x) {
  DF = read.csv(x, header = T, sep = ",")
  DF = DF[!is.na(DF$SPCD),]
  #count unique species numbers for each plot in a specific sampling year
  DF=ddply(DF, .(INVYR,STATECD,COUNTYCD,PLOT,SPCD), summarize, COUNT = length(SPCD))
  return(DF)})

data1 = do.call(rbind, data_all1)


###step 2: get alpha plant species richness
data_all2 = lapply(L2, function(x) {
  DF = read.csv(x, header = T, sep = ",")
  DF = DF[!is.na(DF$VEG_SPCD),]
  CAN = ddply(DF, .(INVYR,STATECD,COUNTYCD,PLOT), summarize, VEG_SPCD = length(unique(VEG_SPCD)))
  return(CAN)})

data2 = do.call(rbind, data_all2)

length(unique(data1$PLOT))#do a check if the previous step is correct
mean(data1$SPECIMEN_COLLECTED)


###step 3: get the geographic coordinates for all plots
data_all3 = lapply(L3, function(x) {
  DF = read.csv(x, header = T, sep = ",")
  DF = DF[!is.na(DF$ELEV),]
  DF = DF[!duplicated(DF[,c('LAT', 'LON', 'ELEV')]),]
  DF = DF[c(5,6,8:9,20:22)]
  return(DF)})
```

```
data3 = do.call(rbind, data_all3)

length(unique(data3$PLOT))
length(unique(data3$COUNTYCD))


###step 4: get total aboveground dry biomass
data_all4 = lapply(L4, function(x) {
  DF = read.csv(x, header = T, sep = ",")
  DF[is.na(DF)] = 0
  DF["biomass"] = 0
  DF$biomass = rowSums(DF[,c("DRYBIO_BOLE", "DRYBIO_TOP","DRYBIO_STUMP",
                           "DRYBIO_SAPLING","DRYBIO_WDLD_SPP")])
  DF = DF[!is.na(DF$biomass),]
  CAN = ddply(DF, .(INVYR,STATECD,COUNTYCD,PLOT), summarize, biomass = mean(biomass))
  return(CAN)})

data4 = do.call(rbind, data_all4)


###step 5: get productivity classes
data_all5 = lapply(L5, function(x) {
  DF = read.csv(x, header = T, sep = ",")
  DF = DF[!is.na(DF$SITECLCD),]
  CAN = ddply(DF, .(INVYR,STATECD,COUNTYCD,PLOT), summarize, SITECLCD = mean(SITECLCD))
  return(CAN)})

data5 = do.call(rbind, data_all5)

nrow(data5)
length(unique(data5$PLOT))


###step 6: merge data
```

```
#get qualified plots
data_SPCD=data1[!duplicated(data1[,c('SPCD')]),]
data_SPCD=data_SPCD[c(2)]
SPCD=merge(data_SPCD,data_SPCD_All, by="SPCD")

dat2=merge(SPCD,data1,by=c("SPCD"))
dat3=ddply(dat2, .(INVYR,STATECD,COUNTYCD,PLOT,Conifers), summarize, total=sum(COUNT))
dat4=ddply(dat3, .(INVYR,STATECD,COUNTYCD,PLOT), summarize, Conifers= Conifers,
ratio=total/sum(total))

#how many compiled plots so far?
nrow(dat4[!duplicated(dat4[,c('INVYR','STATECD','COUNTYCD','PLOT')]),])

#retain conifer-dominated plots
dat5=subset(dat4,Conifers == "Y" & ratio >= 0.7)

#for instance, merge dat5 and data3
Diversity_Tree = merge(dat5,data3,by=c("INVYR","STATECD","COUNTYCD","PLOT"), all = F)
length(unique(Diversity_Tree$PLOT))
anyDuplicated(Diversity_Tree$PLOT, incomparables = FALSE, fromLast = TRUE)

#likewise, carry out conditional merge for data2, data4, and data5 with dat5 (and data1, if
necessary),
#and the new data frames are named as Species_Richness, Biomass, and Productivity, respectively


###step 7: save the compiled data from the FIA
write.csv(Diversity_Tree, "Diversity_Tree.csv", row.names=FALSE)
write.csv(Species_Richness, "Species_Richness.csv", row.names=FALSE)
write.csv(Biomass, "Biomass.csv", row.names=FALSE)
write.csv(Productivity, "Productivity.csv", row.names=FALSE)
```

```
###step 8: calculate tree height for each plot using the annual climatic variable-based niche
model (see Table 2)
#-------------------------------------------------------------------------------------------#
#prior to the following step, it is imperative to calculate virtual tree height for the plots #
#based on their geographic coordinates that have been merged in above                       #
#this calculation was made for all qualified plots before further merge and filtering       #
#-------------------------------------------------------------------------------------------#

###step 9: remove rows with biomass<=0 & hegiht<=0
#for instance for tree diversity data, we do
DF = read.csv("Diversity_Tree.csv",header = T)
DF = DF[apply(DF[c(1)],1,function(z) !any(z<=0)),]
DF = DF[apply(DF[c(5:12)],1,function(z) !any(z<0)),]
write.csv(DF, "Diversity_Tree.csv", row.names=FALSE)

#likewise, carry out similar filtering for other data sets
```

Note S1 Reference list[1] for the source of observed tree heights

Wright J. W., W. A. Lemmien, and J. N. Bright. 1971. Genetic variation in Southern Rocky Mountain white fir. Silvae Genetica 20:148-150.

König, A. 1995. Geographic variation of *Abies grandis*-provenances grown in northwestern Germany. Silvae Genetica 44:248-255.

Xie C. Y., and C. C. Ying. 1993. Geographic variation of grand fir (*Abies grandis*) in the Pacific coast region: 10-year results from a provenance trial. Canadian Journal of Forest Research 23:1065-1072.

Hansen O. K., U. B. Nielsen, Ø. M. Edvardsen, B. Skúlason, and J.-O. Skage. 2004. Nordic provenance trials with *Abies lasiocarpa* and *Abies lasiocarpa* var. *arizonica*: three-year results. Scandinavian Journal of Forest Research 19:112-126.

Xie, C. Y. and C. C. Ying. 1994. Adaptedness of noble fir (*Abies procera* Rehd.) beyond its northern limit. Forest Science 40:412-428.

Rehfeldt, G. E. 1994. Adaptation of *Picea engelmannii* populations to the heterogeneous environments of the Intermountain West. Canadian Journal of Botany 72:1197-1208.

Bongarten, B. C. 1978. Genetic and environmental variation in shoot growth and other traits of blue spruce (*Picea pungens*). PhD thesis, Michigan State University, East Lansing, MI.

Mimura, M., S.N. Aitken. 2007. Adaptive gradients and isolation-by-distance with postglacial migration in *Picea sitchensis*. Heredity 99:224-232.

Ying, C. C. 1997. Effects of site, provenance, and provenance and site interaction in Sitka spruce in coastal British Columbia. Forest Genetics 4:99-112.

Bower, A. D. and S. N. Aitken. 2008. Ecological genetics and seed transfer guidelines for *Pinus albicaulis* (Pinaceae). American Journal of Botany 95:66-76.

Brown A. G., and J. C. Doran. 1985. Variation in growth and branching characteristics of *Pinus attenuata*. Silvae Genetica 34:100-104.

Dow B. D., R. A. Cunningham, and J. M. Krupinsky. 1998. Fifteen-year provenance tests of lodgepole pine (*Pinus contorta*) in North Dakota. Western Journal of Applied Forestry 13:5-11.

Illingworth, K. 1978. Study of lodgepole pine genotype-environment interaction in B.C. In: International Union of Forestry Research Organizations (IUFRO) Joint Meeting of Working Parties: Douglas-fir Provenances, Lodgepole Pine provenances, Sitka Spruce Provenances and *Abies* Provenances, Vancouver, British Columbia, Canada. pp 151-158.

Rehfeldt, G. E., R. J. Hoff, and R. J. Steinhoff. 1984. Geographic patterns of genetic variation in *Pinus monticola*. Botanical Gazette 145:229-239.

[1] The data have been the subject of the quoted publication, [Aitken, S. N. and J. B. Bemmels (2016); Evol. Appl. 9, 271-290], but some data sets used in this study should be directly extracted from the original articles.

Enricci J. A., N. M. Pasquini, O. A. Picco, and V. Mondino. 2000. Provenance trials on *Pinus ponderosa* Douglas ex Lawson in Argentina's Andean Patagonia. Forest Genetic Resources (FAO), vol 1020-4431, No. 28. Food and Agriculture Organization of the United Nations, Rome, Italy.

Read, R.A. 1980. Genetic variation in seedling progeny of Ponderosa pine provenances. Forest Science Monographs 23:1-60.

Sweet, G. B. 1965. Provenance differences in Pacific coast Douglas-fir. Silvae Genetica 14:46-56.

White T. L., and K. K. Ching. 1985. Provenance study of Douglas-fir in the Pacific Northwest region. IV. Field performance at age 25 years. Silvae Genetica 34:84-90.

Cherry, M. L. 1995. Genetic variation in western red cedar (*Thuja plicata* Donn) seedlings. PhD thesis, University of British Columbia, Vancouver, BC, Canada.

Kuser, J. E., and K. K. Ching. 1981. Provenance variation in seed weight, cotyledon number, and growth rate of western hemlock seedlings. Canadian Journal of Forest Research 11: 662-670.

Table S1 Adaptive strategies via ecological filtering in conifer species

focus species	distinct habitat/species features	key environmental factor(s) for native range limits
Abies concolor	grow on high mountains	temperature, rainfall
Abies grandis	grow on a wide variety of sites	-
Abies lasiocarpa	widely distributed	temperature (narrow MAT range), moisture
Abies procera	grow at high elevations	moisture, temperature
Picea engelmannii	widely distributed in humid climates	temperature (narrow MAT range)
Picea pungens	grow at high elevations, well tolerant to drought & low temperature	moisture
Picea sitchensis	grow in a narrow strip along the north Pacific coast (maritime climate)	abundant moisture throughout the year
Pinus albicaulis	grow at high elevations and timberlines with cold, windy, and snowy climates	warm and dry exposure
Pinus attenuata	grow in mild climates	soil texture (dry rocky mountain)
Pinus contorta	adaptive specialist	well adapted to low temperatures, severely restricting photosynthesis within its range
Pinus monticola	adaptive generalist	moisture, temperature
Pinus ponderosa	little tolerance for short growing seasons	soil moisture and summer rainfall
Pseudotsuga menziesii	adaptive specialist	(physiological constraints)
Thuja plicata	adaptive generalist	temperature
Tsuga heterophylla	thrive in humid climates	moisture (rainfall and fog)

Note: primary source of information: Burns and Honkala (1990)[2];
the geographic distribution for study species in North America is given in Figure S1;
MAT denotes mean annual temperature.

[2] Burns, R. M. and B. H. Honkala. 1990. Silvics of North America: I. Conifers. Agriculture Handbook 654, U.S. Dept. of Agriculture, Forest Service, Miscellaneous Publication, Washington, D.C.

Table S2 Other best-fit growth functions and coefficients used in models (Eqn. 2)

Best-fit functions	Parameter estimates	Goodness-of-fit statistics		
		Pseudo R^2	RMSE	AIC
(a) Key annual climatic variable-based models				
$\theta_1 = a_{10} + a_{11}*DD5 + a_{12}*DD\_18 + a_{13}*Eref + \varepsilon_1$<br>$\theta_2 = a_{20} + \underline{a_{21}}*DD\_18 + \varepsilon_2$	$a_{10} \sim \mathcal{N}(3.221379, 0.1636)$, $a_{11} = 0.048781$, $a_{12} = -0.02461$, $a_{13} = -0.15799$<br>$a_{20} \sim \mathcal{N}(0.088676, 0.00318)$, $a_{21} = 0.000118$	0.9702	0.6569	1236.42
$\theta_1 = a_{10} + a_{11}*DD5 + a_{12}*DD\_18 + \varepsilon_1$<br>$\theta_2 = a_{20} + a_{21}*DD5 + \varepsilon_2$	$a_{10} \sim \mathcal{N}(1.476556, 0.2803)$, $a_{11} = 0.038241$, $a_{12} = -0.01199$<br>$a_{20} \sim \mathcal{N}(0.113189, 0.00479)$, $a_{21} = -0.00078$	0.9634	0.7280	1362.01
$\theta_1 = a_{10} + a_{11}*DD5 + a_{12}*DD\_18 + a_{13}*MAT + \varepsilon_1$<br>$\theta_2 = a_{20} + a_{21}*DD5 + \varepsilon_2$	$a_{10} \sim \mathcal{N}(0.44926, 1.5915)$, $a_{11} = 0.036412$, $a_{12} = 0.003837$, $a_{13} = 0.057937$<br>$a_{20} \sim \mathcal{N}(0.1136, 0.00482)$, $a_{21} = -0.00079$	0.9634	0.7285	1363.85
$\theta_1 = a_{10} + a_{11}*DD5 + a_{12}*DD\_18 + \varepsilon_1$<br>$\theta_2 = a_{20} + a_{21}*DD\_18 + \varepsilon_2$	$a_{10} \sim \mathcal{N}(2.097169, 0.1336)$, $a_{11} = 0.015303$, $a_{12} = -0.01824$<br>$a_{20} \sim \mathcal{N}(0.091467, 0.00357)$, $a_{21} = 0.000225$	0.9629	0.7333	1370.99
$\theta_1 = a_{10} + a_{11}*MAT + \varepsilon_1$<br>$\theta_2 = a_{20} + \underline{a_{21}}*MAT + \varepsilon_2$	$a_{10} \sim \mathcal{N}(1.114483, 0.0741)$, $a_{11} = 0.072931$, $a_{20} \sim \mathcal{N}(0.102737, 0.00274)$, $a_{21} = -0.00042$	0.9626	0.7359	1374.27
(b) Key seasonal climatic variable-based models				
$\theta_1 = a_{10} + \underline{a_{11}}*DD\_18\_sp + \underline{a_{12}}*DD\_18\_at + a_{13}*DD5\_sp + \underline{a_{14}}*DD5\_at + \varepsilon_1$<br>$\theta_2 = a_{20} + a_{21}*DD5\_sp + \varepsilon_2$	$a_{10} \sim \mathcal{N}(1.527861, 0.3332)$, $a_{11} = -0.06097$, $a_{12} = 0.020847$, $a_{13} = 0.163741$, $a_{14} = 0.063875$<br>$a_{20} \sim \mathcal{N}(0.107411, 0.00335)$, $a_{21} = -0.00365$	0.9658	0.7038	1322.27
$\theta_1 = a_{10} + a_{11}*DD\_18\_sp + \underline{a_{12}}*DD\_18\_at + \underline{a_{13}}*DD5\_sp + a_{14}*DD5\_at + \varepsilon_1$<br>$\theta_2 = a_{20} + a_{21}*DD5\_at + \varepsilon_2$	$a_{10} \sim \mathcal{N}(1.316765, 0.3779)$, $a_{11} = -0.06057$, $a_{12} = 0.02924$, $a_{13} = 0.021609$, $a_{14} = 0.179212$<br>$a_{20} \sim \mathcal{N}(0.10996, 0.00382)$, $a_{21} = -0.00276$	0.9657	0.7052	1324.81
$\theta_1 = a_{10} + a_{11}*DD\_18\_sp + a_{12}*DD5\_at + \varepsilon_1$<br>$\theta_2 = a_{20} + a_{21}*DD5\_at + \varepsilon_2$	$a_{10} \sim \mathcal{N}(1.633304, 0.2400)$, $a_{11} = -0.04621$, $a_{12} = 0.138723$<br>$a_{20} \sim \mathcal{N}(0.108327, 0.00280)$, $a_{21} = -0.00229$	0.9653	0.7095	1330.34
$\theta_1 = a_{10} + a_{11}*DD\_18\_sp + \varepsilon_1$<br>$\theta_2 = a_{20} + \underline{a_{21}}*DD\_18\_sp + \varepsilon_2$	$a_{10} \sim \mathcal{N}(2.913061, 0.1314)$, $a_{11} = -0.10874$<br>$a_{20} \sim \mathcal{N}(0.090227, 0.00303)$, $a_{21} = 0.000691$	0.9646	0.7161	1340.65
$\theta_1 = a_{10} + a_{11}*DD5\_sp + \varepsilon_1$<br>$\theta_2 = a_{20} + a_{21}*DD5\_sp + \varepsilon_2$	$a_{10} \sim \mathcal{N}(0.694764, 0.0579)$, $a_{11} = 0.378306$<br>$a_{20} \sim \mathcal{N}(0.112725, 0.00207)$, $a_{21} = -0.00502$	0.9640	0.7225	1351.59

(To be continued)

(Continued)

(c) Key climatic variable-based models (annual + seasonal)				
$\theta_1 = a_{10} + a_{11}*DD5 + a_{12}*DD\_18 + a_{13}*Eref + a_{14}*DD5\_sp + \varepsilon_1$<br>$\theta_2 = a_{20} + a_{21}*DD5 + a_{22}*DD5\_sp + \varepsilon_2$	$a_{10} \sim \mathcal{N}(3.632668, 0.3884)$, $a_{11} = -0.08674$, $a_{12} = -0.0223$, $a_{13} = -0.1277$, $a_{14} = 0.631308$<br>$a_{20} \sim \mathcal{N}(0.08487, 0.00527)$, $a_{21} = 0.002157$, $a_{22} = -0.0112$	0.9712	0.6457	1217.10
$\theta_1 = a_{10} + a_{11}*DD\_18 + a_{12}*Eref + a_{13}*MAT + a_{14}*DD5\_sp + \varepsilon_1$<br>$\theta_2 = a_{20} + a_{21}*DD5\_sp + \varepsilon_2$	$a_{10} \sim \mathcal{N}(-3.33195, 1.4677)$, $a_{11} = 0.07526$, $a_{12} = -0.12661$, $a_{13} = 0.347254$, $a_{14} = 0.239011$<br>$a_{20} \sim \mathcal{N}(0.10176, 0.00242)$, $a_{21} = -0.00285$	0.9712	0.6466	1217.85
$\theta_1 = a_{10} + a_{11}*Eref + a_{12}*MAT + a_{13}*DD5\_sp + \varepsilon_1$<br>$\theta_2 = a_{20} + a_{21}*DD5\_sp + \varepsilon_2$	$a_{10} \sim \mathcal{N}(1.559953, 0.1161)$, $a_{11} = -0.11929$, $a_{12} = 0.072827$, $a_{13} = 0.269061$<br>$a_{20} \sim \mathcal{N}(0.10065, 0.00246)$, $a_{21} = -0.00278$	0.9710	0.6482	1219.88
$\theta_1 = a_{10} + a_{11}*DD\_18 + a_{12}*Eref + a_{13}*DD5\_sp + \varepsilon_1$<br>$\theta_2 = a_{20} + a_{21}*DD5 + a_{22}*DD5\_sp + \varepsilon_2$	$a_{10} \sim \mathcal{N}(2.977114, 0.2549)$, $a_{11} = -0.01993$, $a_{12} = -0.13659$, $a_{13} = 0.289361$<br>$a_{20} \sim \mathcal{N}(0.095836, 0.00333)$, $a_{21} = 0.000566$, $a_{22} = -0.00475$	0.9710	0.6486	1221.70
$\theta_1 = a_{10} + a_{11}*DD\_18 + a_{12}*Eref + a_{13}*DD5\_sp + \varepsilon_1$<br>$\theta_2 = a_{20} + a_{21}*DD5\_sp + \varepsilon_2$	$a_{10} \sim \mathcal{N}(2.829451, 0.2332)$, $a_{11} = -0.0197$, $a_{12} = -0.11626$, $a_{13} = 0.279646$<br>$a_{20} \sim \mathcal{N}(0.10054, 0.00246)$, $a_{21} = -0.00281$	0.9709	0.6494	1222.22

N.B. the same note with Table 1 in the main text.
Asymptotical equivalence for AIC and leave-one out (LOO) cross-validation can be referred to Stone (1977)[3].

[3] Stone, M. 1977. An asymptotic equivalence of choice of model by cross-validation and Akaike's criterion. Journal of the Royal Statistical Society. Series B (Methodological) **39**:44-47.

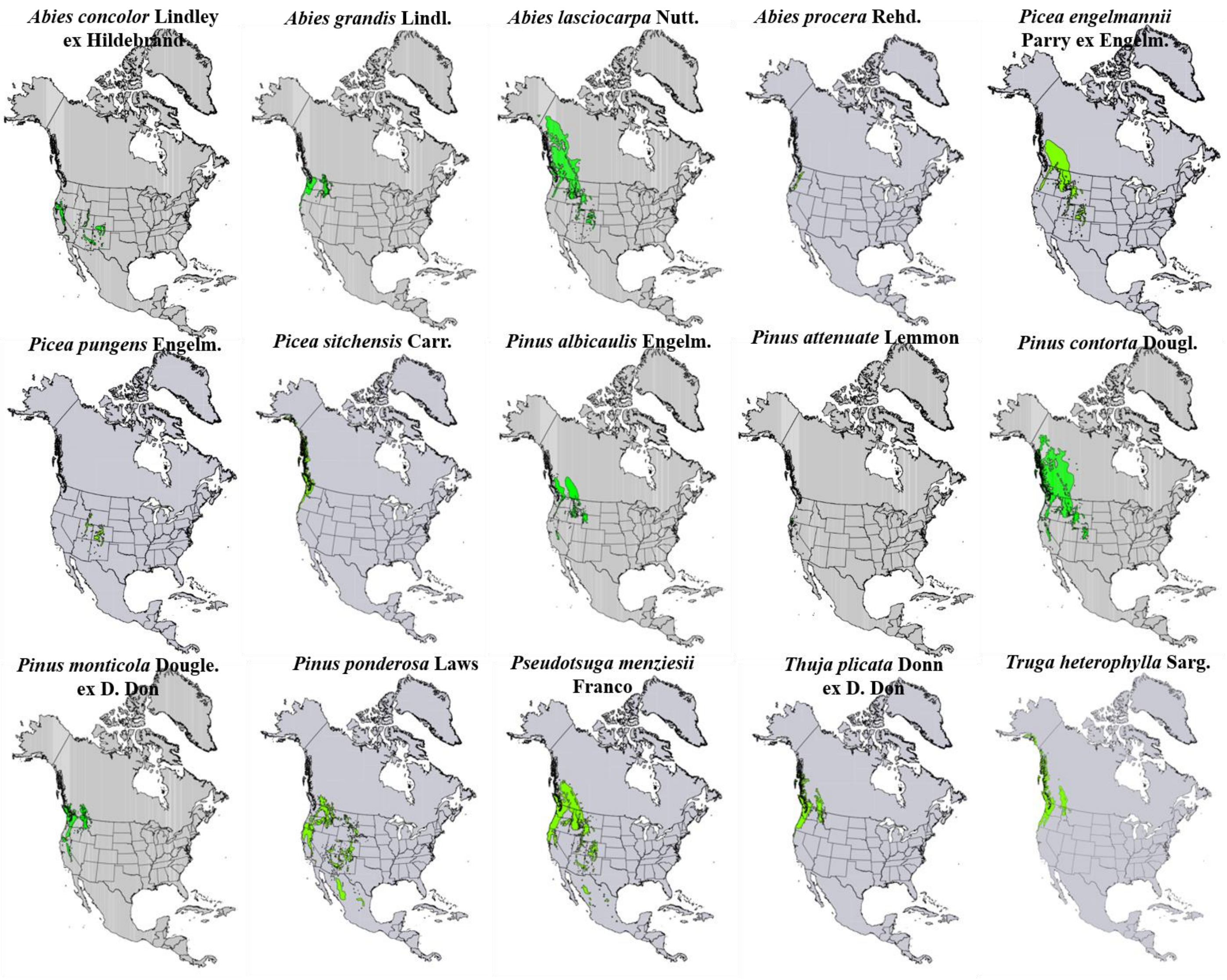

Figure S1 Maps of the ranges of study conifer species in North America (area shaded in green)

Note: maps of the ranges of tree species in North America compiled by Elbert Little and others were digitized for use in USGS vegetation and climate modeling studies ("Atlas of United States trees", https://esp.cr.usgs.gov/data/little/).

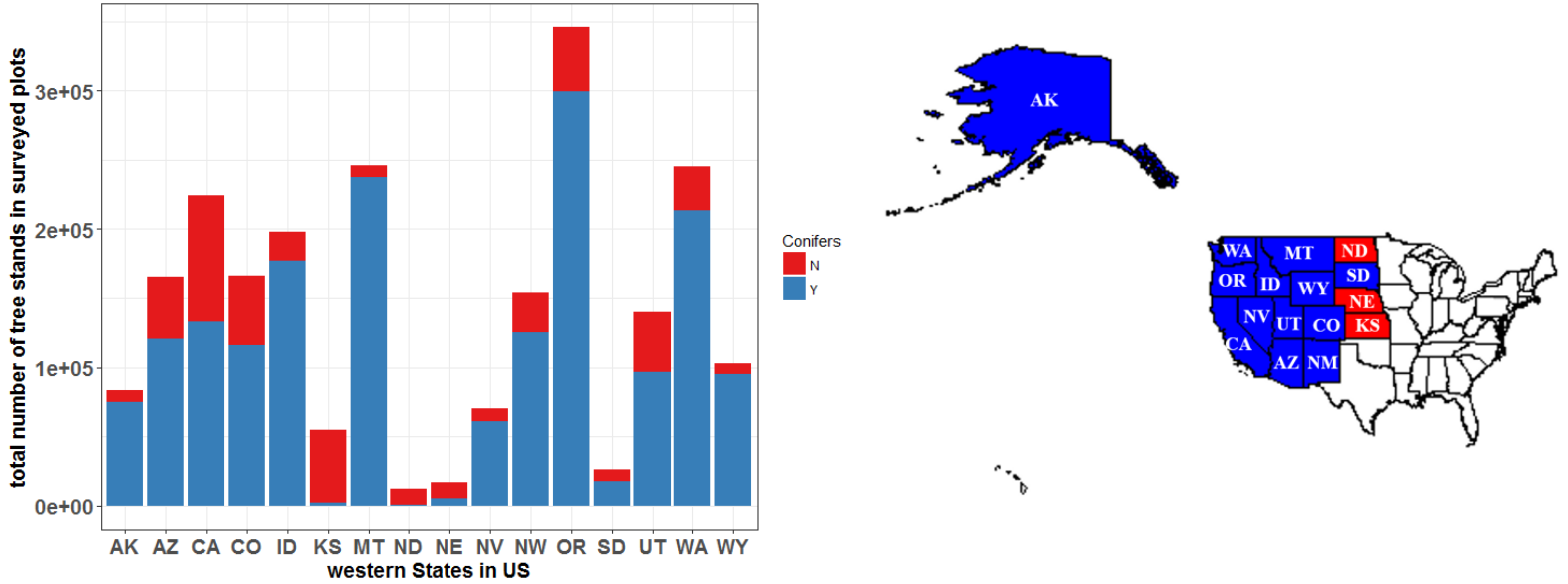

Figure S2 The total number of natural tree stands (conifers vs. non-conifers) in western 16 US States averaged by sampling year

Note: a total number of 80,119 plots are extracted; on the US map, States in blue represent where conifers outnumber other hardwood tree species ("Y" in legend), otherwise, in red ("N" in legend).

Abbreviations of the States: AK (Alaska), WA (Washington), OR (Oregon), CA (California), ID (Idaho), NV (Nevada), MT (Montana), WY (Wyoming), UT (Utah), AZ (Arizona), CO (Colorado), NM (New Mexico), ND (North Dakota), SD (South Dakota), NE (Nebraska), and KS (Kansas).

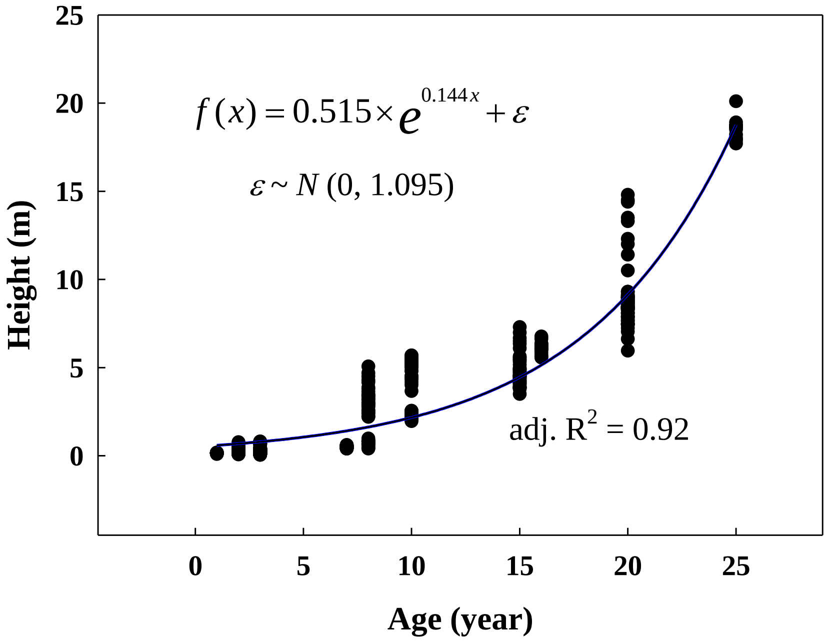

Figure S3 Curve fitting using exponential function for the observed height (population level) - age (under 25) relationship

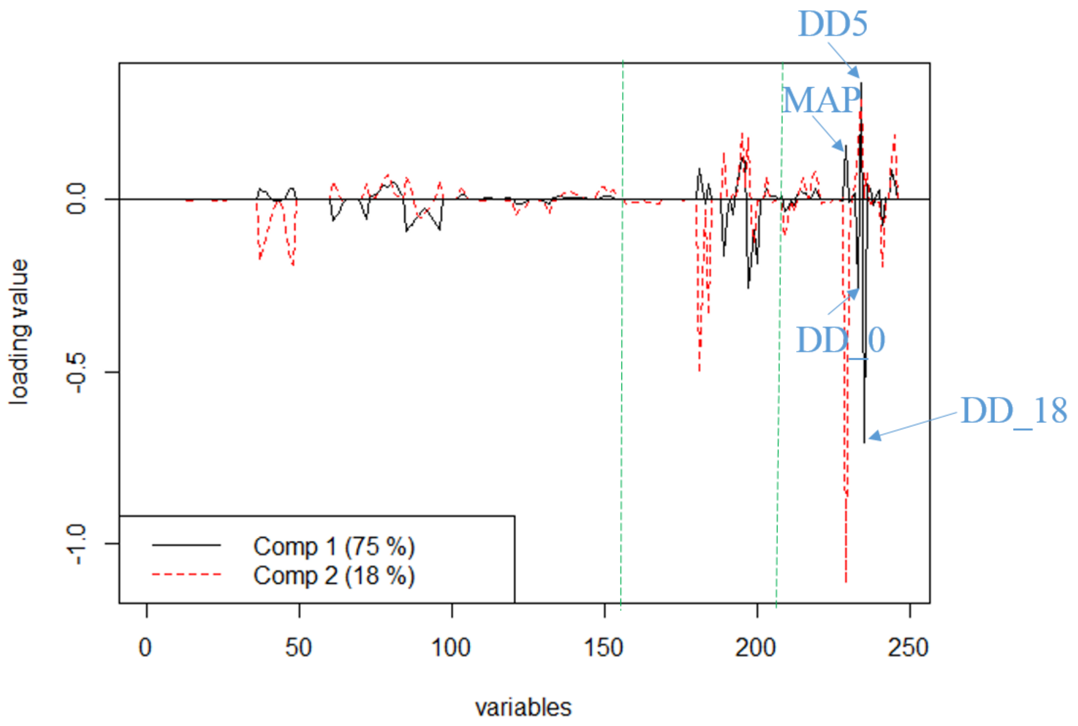

Figure S4 Loadings plot for environmental variables using PLSR

Note: variables, 1-168, 169-224 and 225-247, give monthly, seasonal, annual climatic variables, respectively; the seasonal variables with absolute loading value larger than 0.1 include DD_18_wt/ sp/ at, *DD5_sm*, *DD_0_wt*; acronym annotations for key potential annual selective drivers marked on the graph:

DD_18 (or *_0*): annual degree-days below 18 (or 0) (°C)

DD5: annual degree-days above 5 (°C; growing degree-days or heat sum)

MAP: mean annual precipitation (mm)

The extensions, *sp*/ *sm*/ *at*/ *wt* appended to each variable are abbreviations of four seasons (spring, summer, autumn and winter, respectively)

[N.B. additionally for PLSR, cross-validated RMSEP estimates for first two components give 15.99 and 17.11 for train and test data sets, respectively, indicative of similar prediction errors.]

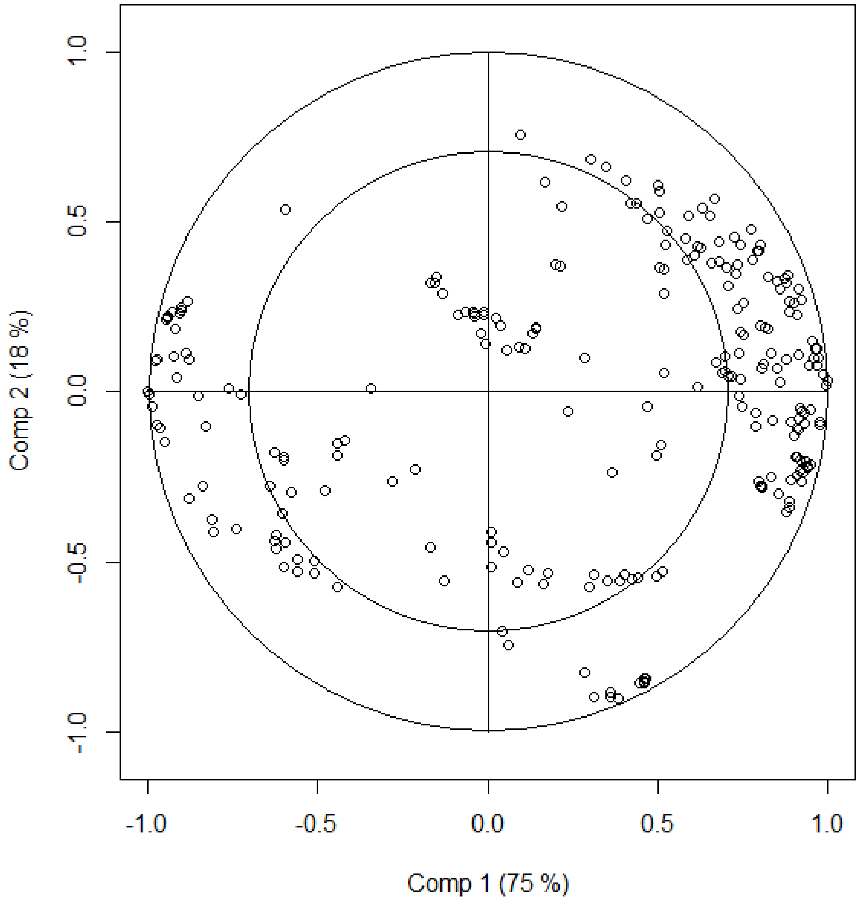

Figure S5 Scores plot for height growth potentials using PLSR

Note: the percentage in parentheses gives the relative amount of height variance explained by each component.

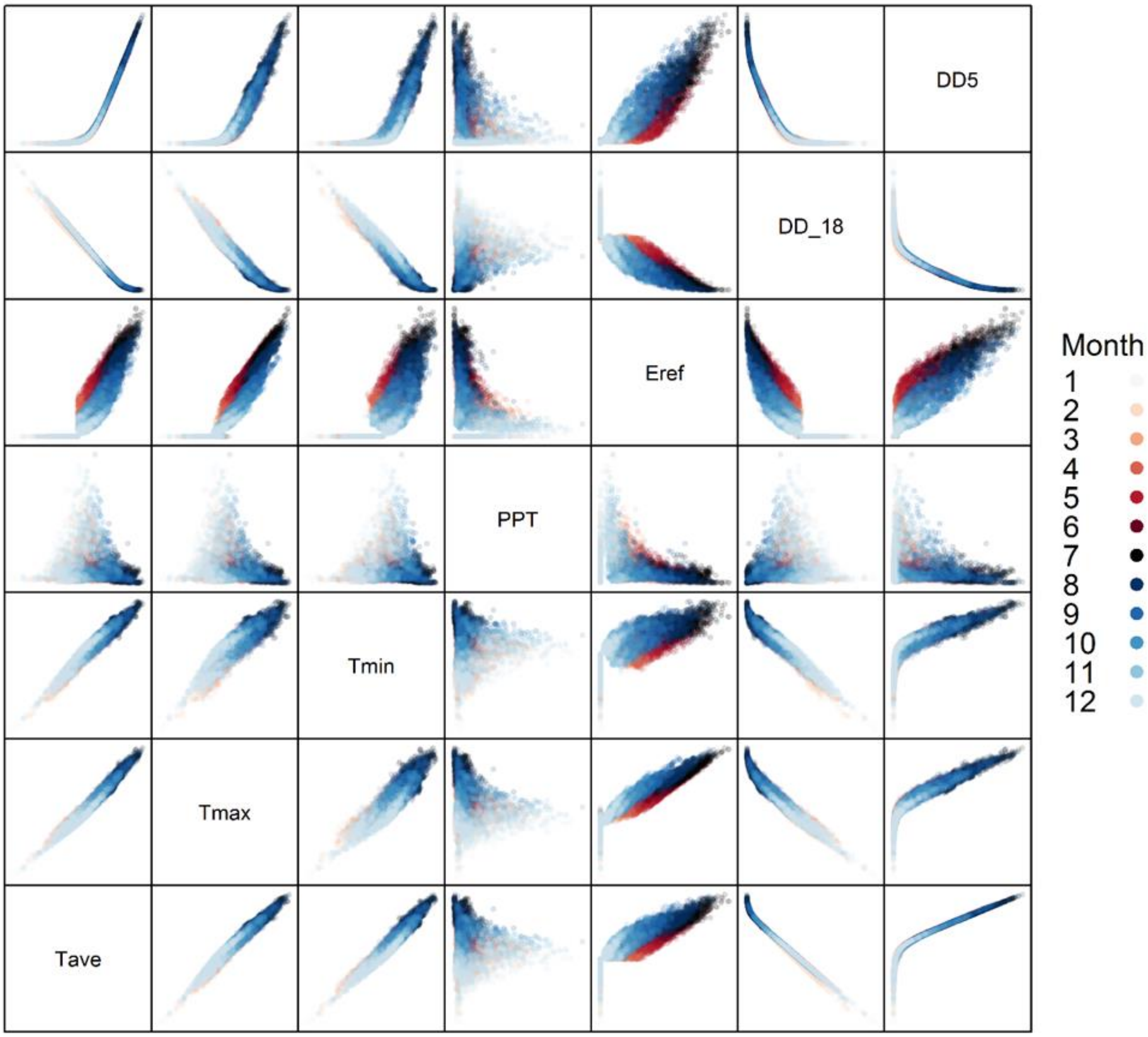

Figure S6 Scatter plot matrix of meteorological time series in 616 study locations
Note: the climate was distinguished between the first and second half of the year with red and blue, respectively; temperature-based variables are almost linearly related and no appreciable pattern between rainfall and any other variables; the relation between evapotranspiration and other variables is different during both halves of the year; the abbreviation and full name for the monthly climatic variables are,
DD5: degree-days above 5 (°C)
DD_18: degree-days below 18 (°C)
Eref: Hargreaves reference evaporation (mm)
PPT: precipitation (mm)
Tmin: mean minimum temperature (°C)
Tmax: mean maximum temperature (°C)
Tave: mean temperature (°C)

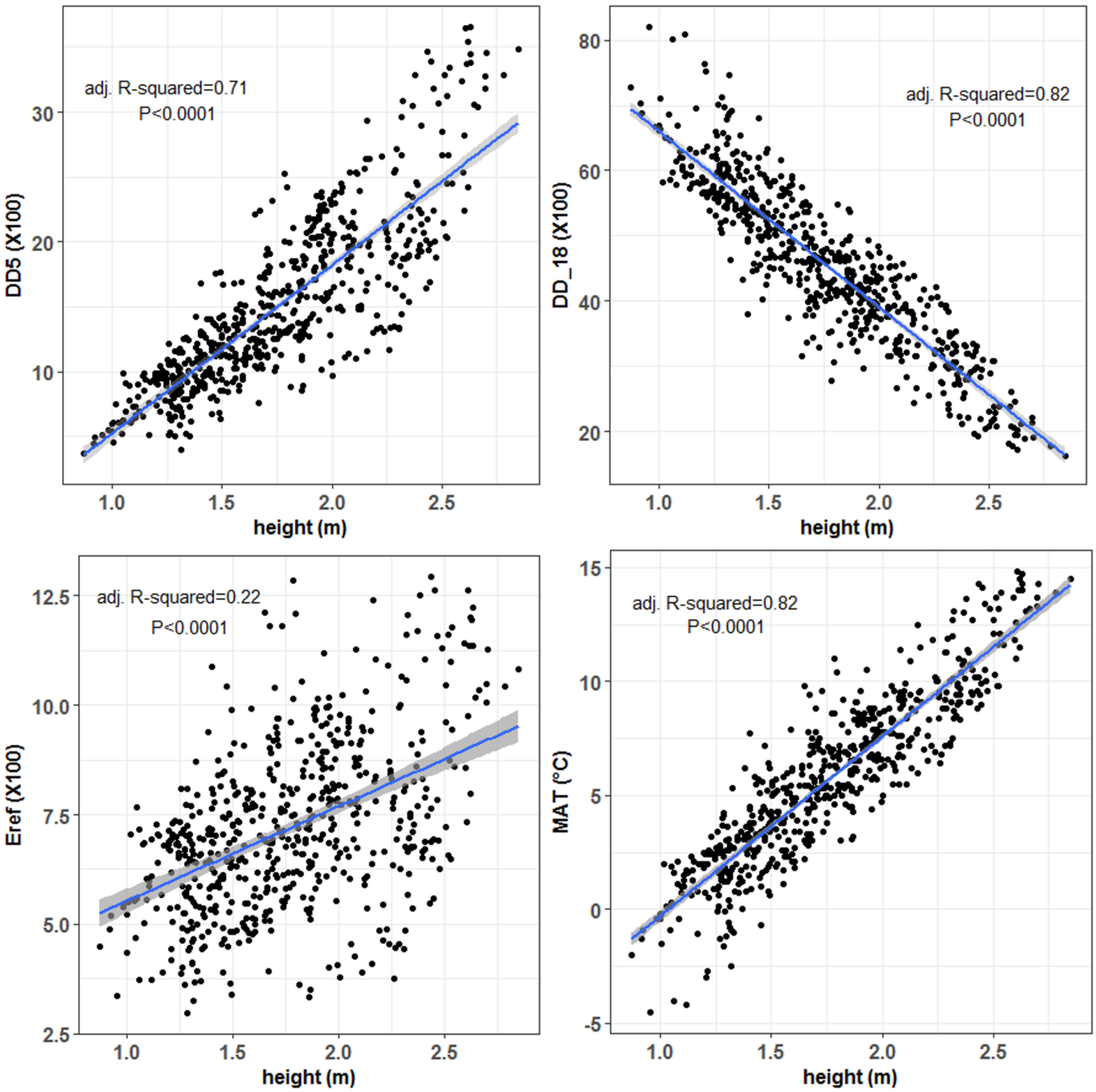

Figure S7 Linear relations of tree height (corrected using Fig. 3A) at 8 with four climatic variables

Note: performance of significant models was displayed with *p* values (*F* tests), adj. R^2 and standard error (shaded grey area).

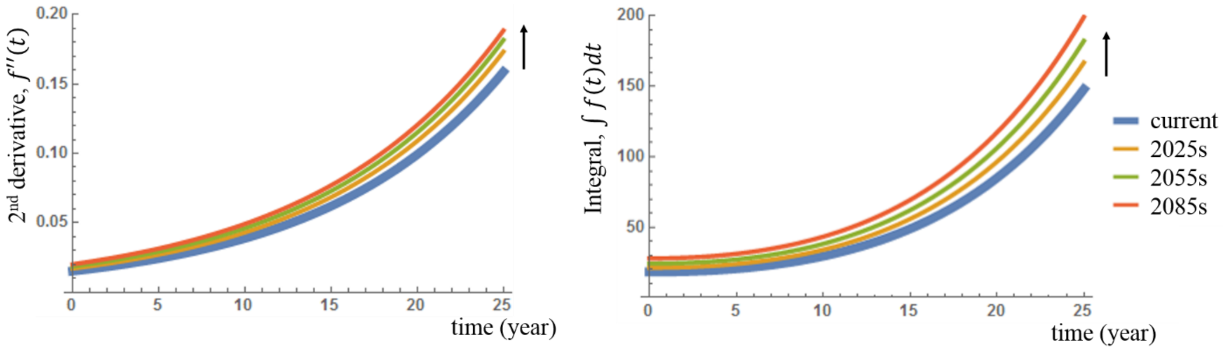

Figure S8 Second derivative and integral for the best-fit model at present and in 2025s, 2055s and 2085s (scenario: RCP8.5)

Note: similar patterns were obtained for scenario RCP4.5, not shown here.

RCP 4.5 and 8.5 scenarios (Rogelj et al. 2016)[4] stand for representative concentration pathways, rising radioactive forcing pathway leading to 4.5 and 8.5 W/m^2 in 2100, respectively. In the RCP 4.5 scenario, temperature increases 2.7 °C (2.1–3.2 °C) and stabilizes before the year 2100 due to the decrease in greenhouse gas emissions (i.e., in consistency with the conditional Intended Nationally Determined Contributions of the Paris Agreement); in the RCP 8.5, temperature increases 4.1 °C (3.1–4.8 °C) until the year 2100 and continue rising, due to a constant increase in radiation rate resulting from the increasing in greenhouse gas concentration (i.e., in consistency with an absence of emissions policies).

[4] Rogelj, J., M. den Elzen, N. Höhne, T. Fransen, H. Fekete, H. Winkler, R. Schaeffer, F. Sha, K. Riahi, and M. Meinshausen. 2016. Paris Agreement climate proposals need a boost to keep warming well below 2 °C. Nature 534:631-639.
